# Diagnostic heterogeneity and healthcare disparities: an underestimated source of bias in atrial fibrillation global burden estimates

**DOI:** 10.1093/europace/euaf114

**Published:** 2025-06-06

**Authors:** Arnaud Bisson, Thibault Lenormand, Laurent Fauchier

**Affiliations:** Service de Cardiologie, Centre Hospitalier Universitaire Trousseau, Avenue de la République, Tours 37044, France; Service de Cardiologie, Centre Hospitalier Universitaire Trousseau, Avenue de la République, Tours 37044, France; Service de Cardiologie, Centre Hospitalier Universitaire Trousseau, Avenue de la République, Tours 37044, France

We read with interest the article by Tan *et al*. on the global burden of atrial fibrillation (AF) and atrial flutter (AFL) from 1990 to 2021, as part of the Global Burden of Disease (GBD) Study 2021.^1^ This comprehensive analysis represents a remarkable effort to delineate temporal and geographical trends in AF/AFL and offers valuable projections. However, we wish to raise several concerns regarding key limitations of the GBD framework, particularly the potential for significant bias arising from variations in diagnostic practices and healthcare accessibility across regions and over time.

AF/AFL diagnosis is highly dependent on the availability of healthcare infrastructure, screening programmes, and physician awareness. In countries with a low socio-demographic index (SDI), limited access to healthcare, shorter life expectancy, and competing health priorities often lead to underdiagnosis, especially at early and potentially treatable stages.^2,3^ This systemic underdetection likely results in an underestimation of the true burden of AF-related complications, such as heart failure, particularly in earlier time periods where health system capacities were even more constrained.

Conversely, in high SDI regions, the proliferation of diagnostic technologies, increased use of implantable devices, implementation of systematic and opportunistic screening strategies, and the widespread availability of novel stroke prevention and rhythm management therapies have led to proactive AF detection.^4–6^ Such diagnostic intensification may explain much of the observed increase in AF/AFL prevalence and related outcomes in these regions in recent years. Importantly, this does not necessarily reflect a true rise in disease incidence but rather enhanced case identification due to improved healthcare delivery.^7,8^

A major limitation of the current study is the lack of detailed information on the types of AF screening methods, diagnostic criteria, and healthcare delivery models used in each country and across different time periods. Without accounting for these critical variables, the analyses are prone to significant bias, limiting the validity of direct regional comparisons or temporal trends. As acknowledged in prior reports, including the World Heart Federation Roadmap on Atrial Fibrillation, addressing health inequities and disparities in AF detection remains a formidable challenge.^3^

We commend the authors for their extensive work but caution that these methodological limitations must be considered when interpreting the study findings. Future iterations of GBD analyses should aim to incorporate granular, country-level data on diagnostic strategies and healthcare accessibility to provide a more accurate and equitable depiction of the global AF/AFL burden.
